# Near infrared photoimmunotherapy with avelumab, an anti-programmed death-ligand 1 (PD-L1) antibody

**DOI:** 10.18632/oncotarget.12410

**Published:** 2016-10-03

**Authors:** Tadanobu Nagaya, Yuko Nakamura, Kazuhide Sato, Toshiko Harada, Peter L. Choyke, James W. Hodge, Jeffrey Schlom, Hisataka Kobayashi

**Affiliations:** ^1^ Molecular Imaging Program, Center for Cancer Research, National Cancer Institute, National Institutes of Health, Bethesda, Maryland 20892, United States of America; ^2^ Laboratory of Tumor Immunology and Biology, Center for Cancer Research, National Cancer Institute, National Institutes of Health, Bethesda, Maryland 20892, United States of America

**Keywords:** near infrared photoimmunotherapy, PD-L1, lung cancer

## Abstract

Near Infrared-Photoimmunotherapy (NIR-PIT) is a highly selective tumor treatment that employs an antibody-photo-absorber conjugate (APC). Programmed cell death protein-1 ligand (PD-L1) is emerging as a molecular target. Here, we describe the efficacy of NIR-PIT, using fully human IgG_1_ anti-PD-L1 monoclonal antibody (mAb), avelumab, conjugated to the photo-absorber, IR700DX, in a PD-L1 expressing H441 cell line, papillary adenocarcinoma of lung. Avelumab-IR700 showed specific binding and cell-specific killing was observed after exposure of the cells to NIR *in vitro*. In the *in vivo* study, avelumab-IR700 showed high tumor accumulation and high tumor-background ratio. Tumor-bearing mice were separated into 4 groups: (1) no treatment; (2) 100 μg of avelumab-IR700 i.v.; (3) NIR light exposure only, NIR light was administered; (4) 100 μg of avelumab-IR700 i.v., NIR light was administered. Tumor growth was significantly inhibited by NIR-PIT treatment compared with the other groups (*p* < 0.001), and significantly prolonged survival was achieved (*p* < 0.01 vs other groups). In conclusion, the anti-PD-L1 antibody, avelumab, is suitable as an APC for NIR-PIT. Furthermore, NIR-PIT with avelumab-IR700 is a promising candidate of the treatment of PD-L1-expressing tumors that could be readily translated to humans.

## INTRODUCTION

Tumor cells express a variety of surface antigens, collectively known as inhibitory checkpoints, that have the effect of creating an immunosuppressive environment that allows tumor cells to escape immune surveillance. The recent Food and Drug Administration (FDA) approvals of several immune checkpoint inhibitors constitute a major advance in the immunotherapy of malignant tumors. Several monoclonal antibodies (mAbs) directed against programmed cell death protein-1 ligand (PD-L1) and PD-1 have demonstrated clinical benefit in patients with melanoma, Hodgkin's lymphoma, lung and bladder carcinomas, and several other tumor types [1–5]. The mode of action of these anti-PD-1/PD-L1 mAbs is only to inhibit the interaction between PD-1 on immune cells and PD-L1 on tumor cells, thus reducing or eliminating immunosuppressive signals, and leading to enhanced immune cell activation. These fully human or humanized mAbs are either of the IgG4 isotype, which does not mediate antibody dependent cell-mediated cytotoxicity (ADCC), or of the IgG1 isotype and specifically engineered to induce ADCC activity. MSB0010718C (designated avelumab) is a fully human IgG_1_ anti-PD-L1 mAb with potential ADCC properties and is currently in several clinical trials [6]. Despite promising early results, the treatment only with these mAbs has not been successful in all patients.

PD-L1 is expressed in various types of normal cells, including placenta, pancreatic islet cells, mesenchymal stem cells and immune cells [7], but it is overexpressed in many cancers. The overexpression of PD-L1 on cancer cells leads to suppressed T-cell activation, and unimpeded tumor growth [7]; thus, high tumor PD-L1 expression is associated with poor prognosis [8, 9]. Viewed in another way, overexpression of PD-L1 on a tumor signals an aggressive phenotype and is a potential target for the delivery of other types of molecular therapy. Near infrared photoimmunotherapy (NIR-PIT) is a newly developed cancer treatment that employs a targeted monoclonal antibody-photo-absorber conjugate (APC) [10]. The photo-absorber, IRDye700DX (IR700, silica-phthalocyanine dye), is a highly hydrophilic dye, differentiating it from prior hydrophobic dyes used in photodynamic therapy (PDT). A first-in-human Phase 1 trial of NIR-PIT with the APC targeting epidermal growth factor receptor (EGFR) in patients with inoperable head and neck cancer was approved by the US FDA, and is underway (https://clinicaltrials.gov/ct2/show/NCT02422979).

NIR-PIT has been shown to be effective with a variety of different antibodies but has not been previously tested with anti-PD-L1 antibodies [10–15]. In this study, we investigated avelumab-IR700 as a candidate APC for NIR-PIT. Using a PD-L1 expressing H441 cell line, papillary adenocarcinoma of lung, *in vitro* tumor binding, *in vivo* tumor accumulation and intratumoral distribution were evaluated. NIR-PIT was then performed with avelumab-IR700 *in vitro* and in a tumor-bearing mouse model *in vivo*.

## RESULTS

### *In vitro* characterization of H441 cell line

**Figure 1:**
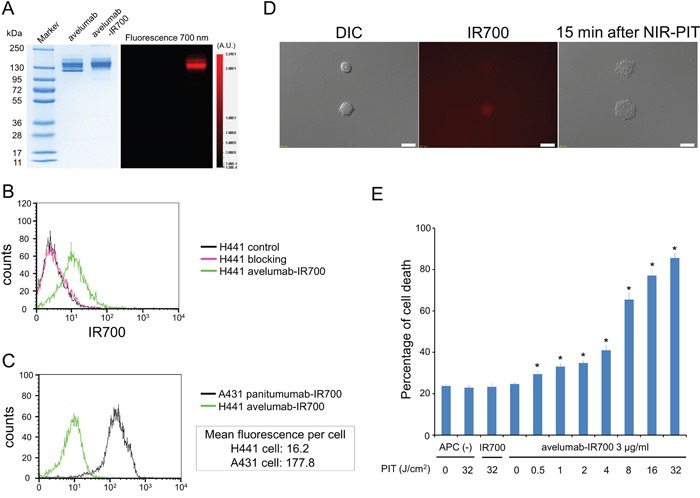
Confirmation of PD-L1 expression as a target for NIR-PIT in H441 cells, and evaluation of *in vitro* NIR-PIT **A.** Validation of avelumab-IR700 by SDS-PAGE (left: Colloidal Blue staining, right: fluorescence). Diluted avelumab was used as a control. **B.** Expression of PD-L1 in H441 cells was examined with FACS. After 6 h of avelumab-IR700 incubation, H441 cells showed high fluorescence signal. **C.** Expression of PD-L1 in H441 cell was estimated by expression of EGFR in A431 cell using FACS. Mean fluorescence of H441 cell with avelumab-IR700 was 16.2, on the other hand the mean fluorescence of A431 cell with panitumumab-IR700 was 177.8. **D.** Differential interference contrast (DIC) and fluorescence microscopy images of H441 cells after incubation with avelumab-IR700 for 6 h. High fluorescence intensities were shown in H441 cells. Necrotic cell death was observed upon excitation with NIR light (after 15min). Scale bars = 20 μm. **E.** Membrane damage of cells induced by PIT was measured with the dead cell count using PI staining, which increased in a light dose dependent manner (n = 5, ^*^p < 0.01, vs. untreated control, by Student's t test).

As defined by SDS-PAGE, the band of avelumab-IR700 was almost the same molecular weight as the non-conjugated control mAb, and fluorescence intensity was identical (Figure 1A). After a 6 h incubation with avelumab-IR700, H441 cells showed high fluorescence signal, which was confirmed with flow cytometry and fluorescence microscopy (Figure 1B, 1D). On the other hand, fluorescence in H441 cells was completely blocked by adding excess avelumab, indicating that avelumab-IR700 specifically binds to the PD-L1 on H441 cells. In addition, to estimate PD-L1 expression level of a H441 cell, mean fluorescence was calculated. Mean fluorescence of H441 cells with avelumab-IR700 was 16.2, on the other hand the mean fluorescence of A431 cells which have the similar size of H441 cells, with panitumumab-IR700 was 177.8 (Figure 1C). Because an A431 cell express approximately 1.5×10^6^ EGFR molecules per cell [16], it was suggested that a H441 cell express approximately 1.4×10^5^ PD-L1 ligands on the cell surface.

### *In vitro* NIR-PIT

Immediately after exposure, NIR light induced cellular swelling, bleb formation, and rupture of vesicles representing necrotic cell death (Supplementary Video). Most of these morphologic changes were observed within 15 min of light exposure (Figure 1D), indicating rapid induction of necrotic cell death. Based on incorporation of propidium iodide (PI), percentage of cell death increased in a light dose dependent manner (Figure 1E). Over 80% of H441 cells died when exposed to 32 J of NIR light. There was no significant cytotoxicity associated with IR700 dye alone with NIR light, with NIR light alone in the absence of APC and with APC alone without NIR light.

### *In vivo* fluorescence imaging studies

**Figure 2:**
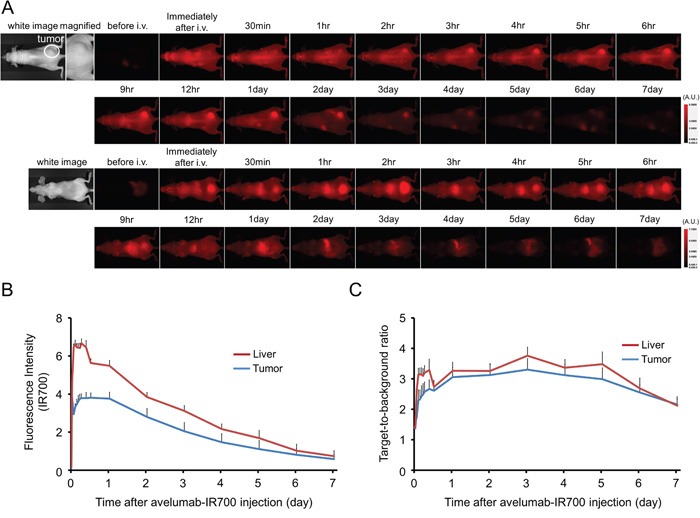
*In vivo* fluorescence imaging of H441tumor **A.** *In vivo* avelumab-IR700 fluorescence real-time imaging of tumor-bearing mice (right dorsum). The tumor showed high fluorescence intensity after injection and the intensity was gradually decreased over days. Most of the excess agent was excreted to the urine immediately after injection. **B.** Quantitative analysis of IR700 intensities in tumors and livers (n = 10). The IR700 fluorescence intensity of tumor and liver shows high intensities within 1 day after APC injection but this decreases gradually over days. **C.** Quantitative analysis of TBR in tumors and livers (n = 10). TBR of tumor and liver shows high immediately after APC injection, then the TBR did not change over days.

The fluorescence intensity of avelumab-IR700 in H441 tumor shows high intensities within 1 day after APC injection but this decreases gradually over the following days (Figure 2A, 2B). On the other hand, target-to-background ratio (TBR) of avelumab-IR700 in tumor and liver is high immediately after APC injection, following which the TBR did not change for several days (Figure 2C). TBR of avelumab-IR700 was high in tumor due to specific avelumab binding to PD-L1 expressing H441 cells, while TBR was supposed to be high in liver due to non-specific accumulation of avelumab-IR700 conjugate. To obtain the maximal therapeutic effect, the tumor fluorescence caused by binding of the APC should be high in tumor and low in background. Tumor fluorescence was high after APC injection, while fluorescence signal of background including liver decreased beginning 12 hours after APC injection. Thus, we used 1 day of incubation with APC to get the maximal difference between tumor and background normal tissue.

### *In vivo* NIR-PIT

**Figure 3:**
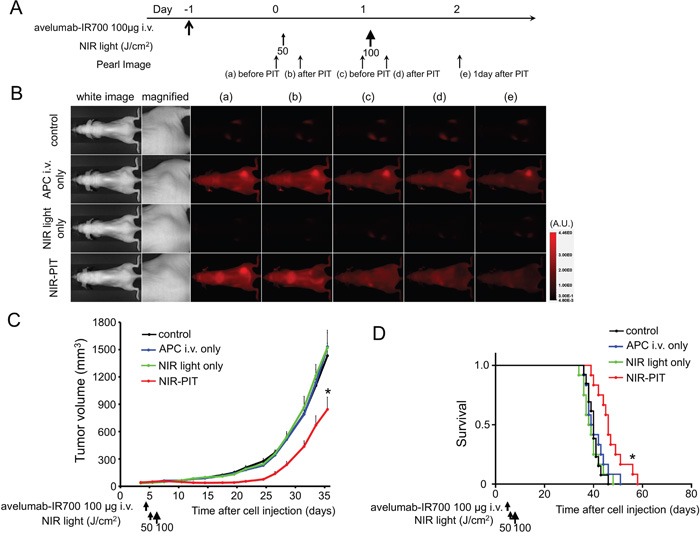
*In vivo* effect of NIR-PIT for H441 tumor **A.** NIR-PIT regimen. Fluorescence images were obtained at each time point as indicated. **B.** *In vivo* fluorescence real-time imaging of tumor-bearing mice in response to NIR-PIT. The tumor treated by NIR-PIT showed decreasing IR700 fluorescence after NIR-PIT. **C.** Tumor growth was significantly inhibited in the NIR-PIT treatment groups (n ≧ 10, *p < 0.001 vs other groups, Bonferroni's test with ANOVA). **D.** Significantly prolonged survival was observed in the NIR-PIT treatment group (n ≧ 10, *p < 0.01 vs other groups, by Log-rank test).

The treatment and imaging regimen is shown in Figure 3A. One day after injection of avelumab-IR700, the tumors showed higher fluorescence intensity than did the tumor with no APC. After exposure to 50 J/cm^2^ of NIR light, IR700 tumor fluorescence signal decreased due to dying cells and partial photo-bleaching, while the IR700 fluorescence did not change for up to 2 days in tumors receiving avelumab-IR700 but no NIR light (Figure 3B). Tumor growth was significantly inhibited in the NIR-PIT treatment group compared with the other groups (p < 0.001) (Figure 3C), and significantly prolonged survival was achieved in the NIR-PIT group (p < 0.01 vs other groups) (Figure 3D). No significant therapeutic effect was observed in the control groups, including those receiving APC only or in mice receiving NIR light only. There was no skin necrosis or toxicity attributable to the APC in any group.

### Histological analysis

**Figure 4:**
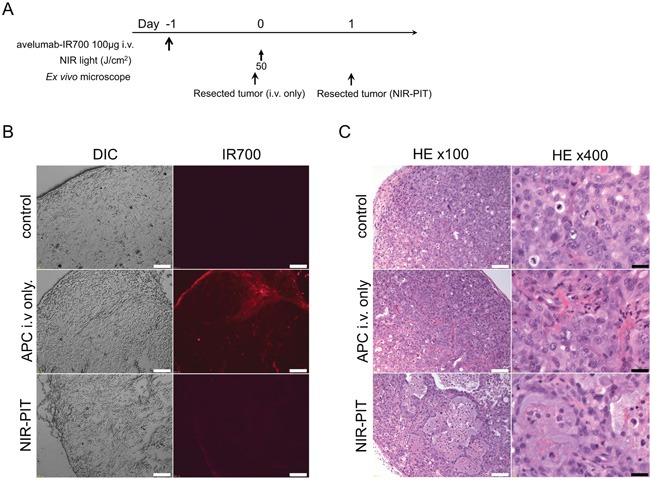
*In vivo* histological fluorescence distribution and histological NIR-PIT effect **A.** The regimen of NIR-PIT. **B.** Differential interference contrast (DIC) and fluorescence microscopy images of H441 tumor xenografts. Fluorescence intensity is shown in H441 cells 24 h after injection of avelumab-IR700, but the fluorescence disappears 24 h after NIR-PIT. Scale bars = 100 μm. **C.** Resected tumor stained with H&E. A few scattered clusters of damaged tumor cells are seen within a background of diffuse cellular necrosis and micro-hemorrhage after NIR-PIT, while no obvious damage was observed after avelumab-IR700 alone with NIR light. White scale bars = 100 μm. Black scale bars = 20 μm.

The treatment and imaging regimen is shown in Figure 4A. Fluorescence intensities of H441 cells 24 h after avelumab-IR700 were compared with control tumor. Almost all fluorescence disappeared 24 h after NIR-PIT (Figure 4B). Hematoxylin and eosin (H&E) staining of NIR-PIT treated H441 tumors revealed diffuse necrosis and micro-hemorrhage, with scattered clusters of live but damaged tumor cells, while no obvious damage was observed in the tumor receiving only avelumab-IR700 but no light (Figure 4C).

## DISCUSSION

Several antibodies against PD-1/PD-L1 have recently been developed for clinical application. These mAbs were shown in phase I/II trials to induce a 30% to 50% response in several cancer types [17]. Recent phase II and phase III studies supported the request for an accelerated approval for many cancers [18–21]. Avelumab is a fully human IgG_1_ anti-PD-L1 mAb with potential ADCC properties and is currently in a clinical trial [6]. The conjugate avelumab-IR700 achieved adequate tumor TBRs as shown in Figure 2, indicating that it may be practical for clinical application during surgical or endoscopic procedures because of its high TBR on the PD-L1 expressing tumors. Efficient binding and distribution of the antibody in the tumor are important for APCs to be effective as agents for NIR-PIT. This also holds for antibody-toxin or antibody-drug conjugates since, to be effective, the drugs and toxins must be internalized after cell binding. Our results showed that avelumab bound to PD-L1 specifically and was internalized within 6 hours of incubation in PD-L1 expressing cancer cells. These results suggest that avelumab has favorable characteristics for an antibody-drug conjugate.

The conjugate avelumab-IR700 proved to be an effective agent for treating a PD-L1 expressing tumor model with NIR-PIT. NIR-PIT with avelumab-IR700 led to rapid cell death *in vitro* and tumor growth reduction and survival improvement *in vivo*. Thus, avelumab-IR700 could be an effective platform for NIR-PIT in PD-L1 expressing tumors. From the pharmacokinetic point of view, IR700 conjugation minimally alters the pharmacokinetics of the antibody due to the small size and hydrophilic nature of the IR700 dye. APCs can be synthesized from virtually any antibody; therefore, NIR-PIT could apply to numerous target molecules across a broad range of tumor types [10–15].

We chose a therapeutic regimen with a single injection of the conjugate and two, not one, light exposures. Rapid and massive cancer cell killing adjacent to tumor vessels with NIR-PIT leads to an immediate increase in vascular permeability. The delivery of various nano-sized or macromolecular drugs into a NIR-PIT treated tumor increases up to 24-fold compared with that in a control non-treated tumor immediately after the initial NIR light exposure that is known as the super enhanced permeability and retention (SUPR) effect [22–24] induced by NIR-PIT. After the first NIR-PIT, circulating APCs more greatly and deeply permeate into the extravascular space of NIR-PIT treated tumors than into non-treated tumors and bind homogeneously to survived cancer cells due to the SUPR effect. Thus, second light exposure was performed for making NIR-PIT even more effective [25].

In cancer therapy, anticancer drugs often fail because heterogeneous vascularity, increased interstitial pressure and other structural barriers imposed by the extracellular matrix prevent the drug from reaching its target in sufficient concentrations to be effective [26, 27]. Moreover, naturally occurring tumors usually are phenotypically and functionally heterogeneous [28, 29]. Repeated NIR-PIT with additional various types of APC or delivery of higher doses of non-targeted anticancer drugs by taking advantage of the SUPR effect are good strategies to improve the therapeutic effect of cancer [24].

NIR-PIT shows highly target-specific cytotoxicity, and NIR light can be easily applied to superficial tumors, such as PD-L1 expressing melanoma. On the other hand, an obvious limitation of NIR-PIT for PD-L1 expressing tumors, is the inability to deliver NIR light to the tumor located deep in the tissue. Skin, fat and other organs will absorb NIR light before it reaches the tumor. There are several potential solutions to this problem. For instance, NIR light could be delivered to a tumor while the tissues are still exposed after a best surgical resection, thus treating residual tumor. Alternatively, light could be administered endoscopically, via a bronchoscope or thoracoscope in the case of lung cancer or via a cystoscope in the case of bladder cancer. A third possibility is to introduce light fibers interstitially into tumors using needle trocars. Such procedures have been proposed in the past with PDT; however, we believe that NIR-PIT would be much more effective with lower toxicity than PDT [30, 31].

An alternative cell-selective cancer therapy with the use of light is the fluorescence-guided ultraviolet C (UVC) irradiation of selectively-labeled tumor cells with fluorescence protein which was transfected using adenovirus and the efficacy of this therapy was previously determined in various tumors [30, 32–34]. However, the wavelength of UVC is shorter than that of NIR, therefore, UVC light does not penetrate deep into tissue. Furthermore, this therapy requires virus-mediated transfection of fluorescence protein gene to cancer cells *in vivo*. Therefore, we think NIR-PIT would be technically simple and easy.

This study has several limitations. We performed one injection of the APC with two exposures of light in order to perform a proof-of-principle study for demonstrating that NIR-PIT with anti-PD-L1 APC was effective to treat PD-L1-expressing cancers. Clearly, repeated dosing of the APC with repeated light exposure is likely to increase effectiveness. For instance, EGFR-targeted NIR-PIT in an appropriate model with a repeated regimen of APC and light dosing was reported to improve the therapeutic effect [25, 35]. It would be desirable to extend these studies to multiple doses of the APC and light. This study was also done in an immune-incompetent mouse model. As the immune effects of NIR-PIT are currently unknown it is difficult to know whether this will be of benefit to patients, although it is anticipated that it will augment the therapeutic effect because it is known that cytokines such as interferon γ induce the expression of PD-L1 in different types of tumor cells [36–38]. Increased expression of PD-L1 would further increase the effect of the NIR-PIT through increased numbers of antibody conjugated IR700 molecules on the cell surface. Finally, subcutaneously-growing human tumors in mice do not sufficiently represent clinical cancer. To clarify the pre-clinical effect, superior tumor models such as surgically orthotopic implantation tumor models are better than xenografted tumor models [39–41], yet surgical orthotopic implant requires highly trained surgical skills. Therefore, in this proof-of-principle study of NIR-PIT targeting PD-L1, we chose a simple subcutaneous xenograft tumor model.

## CONCLUSIONS

Avelumab, a fully human anti-PD-L1 mAb, showed accumulation in PD-L1-expressing cancer cells. NIR-PIT using avelumab-IR700 can induce significant therapeutic responses after only a single injection of the conjugate and two light exposures in a PD-L1-expressing animal tumor model. Thus, NIR-PIT utilizing PD-L1 as the targeting antigen for the APC might be successful in treating cancers that have not previously responded to the naked antibody alone.

## MATERIALS AND METHODS

### Reagents

Water soluble, silica-phthalocyanine derivative, IRDye 700DX NHS ester was obtained from LI-COR Biosciences (Lincoln, NE, USA). A fully human anti-PD-L1 mAb (MSB0010718C; designated avelumab) was kindly provided by EMD Serono. Panitumumab, a fully humanized IgG_2_ mAb directed against EGFR, was purchased from Amgen (Thousand Oaks, CA, USA). All other chemicals were of reagent grade.

### Synthesis of IR700-conjugated avelumab and panitumumab

Conjugation of dyes with mAb was performed according to a previous report [10]. In brief, avelumab (1.0 mg, 6.7 nmol) or panitumumab (1.0 mg, 6.8 nmol) was incubated with IR700 NHS ester (65.1 μg, 33.3 nmol for avelumab, 66.8 μg, 34.2 nmol for panitumumab) in 0.1 M Na_2_HPO_4_ (pH 8.6) at room temperature for 1 h. The mixture was purified with a Sephadex G25 column (PD-10; GE Healthcare, Piscataway, NJ, USA). The protein concentration was determined with Coomassie Plus protein assay kit (Thermo Fisher Scientific Inc, Rockford, IL, USA) by measuring the absorption at 595 nm with UV-Vis (8453 Value System; Agilent Technologies, Santa Clara, CA, USA). The concentration of IR700 was measured by absorption at 689 nm to confirm the number of fluorophore molecules per mAb. The synthesis was controlled so that an average of two IR700 molecules was bound to a single antibody. As a quality control for the conjugate, we performed sodium dodecyl sulfate-polyacrylamide gel electrophoresis (SDS-PAGE). Conjugate was separated by SDS-PAGE with a 4-20% gradient polyacrylamide gel (Life Technologies, Gaithersburg, MD). A standard marker (Crystalgen Inc., Commack, NY) was used as a protein molecular weight marker. After electrophoresis at 80 V for 2.5 h, the gel was imaged with a Pearl Imager (LI-COR Biosciences, Lincoln, Nebraska, USA) using a 700 nm fluorescence channel. We used diluted avelumab as non-conjugated control. The gel was stained with Colloidal Blue staining to determine the molecular weight of conjugate.

### Cell culture

H441, papillary adenocarcinoma of lung with expression of human PD-L1, was used for NIR-PIT. A431 cell expressing EGFR was used to estimate the number of PD-L1 ligands on a H441 cell because both cells have a similar size. Cells were grown in RPMI 1640 (Life Technologies, Gaithersburg, MD, USA) supplemented with 10% fetal bovine serum and 1% penicillin/streptomycin (Life Technologies) in tissue culture flasks in a humidified incubator at 37°C in an atmosphere of 95% air and 5% carbon dioxide.

### Flow cytometry

To verify *in vitro* avelumab-IR700 binding, fluorescence from cells after incubation with APC was measured using a flow cytometer (FACS Calibur, BD BioSciences, San Jose, CA, USA) and CellQuest software (BD BioSciences). H441 cells (2 × 10^5^) were seeded into 12 well plates and incubated for 24 h. Medium was replaced with fresh culture medium containing 3 μg/ml of avelumab-IR700 and incubated for 6 h at 37°C. To validate the specific binding of the conjugated antibody, excess antibody (100 μg) was used to block 10 μg of APCs.

To estimate the number of PD-L1 ligands on a H441 cell *in vitro*, fluorescence from cells after incubation with APC was also measured using a flow cytometer. Similarly sized H441 cells (2 × 10^5^) and A431 cells (2 × 10^5^) were seeded into 12 well plates and incubated for 24 h. Medium was replaced with fresh culture medium containing 10 μg/ml of avelumab-IR700 for H441 cells or panitumumab-IR700 for A431 cells and incubated for 2 h at 37°C. Mean fluorescence was calculated using the CellQuest software.

### Fluorescence microscopy

To detect the antigen specific localization and effect of NIR-PIT, fluorescence microscopy was performed (BX61; Olympus America, Inc., Melville, NY, USA). Ten thousand cells were seeded on cover-glass-bottomed dishes and incubated for 24 h. Avelumab-IR700 was then added to the culture medium at 3 μg/ml and incubated for 6 h at 37°C. After incubation, the cells were washed with phosphate buffered saline (PBS). The filter set to detect IR700 consisted of a 590-650 nm excitation filter, a 665-740 nm band pass emission filter. Transmitted light differential interference contrast (DIC) images were also acquired.

### *In vitro* NIR-PIT

The cytotoxic effects of NIR-PIT with avelumab-IR700 were determined by flow cytometric propidium iodide (PI) (Life Technologies) staining, which can detect compromised cell membranes. Two hundred thousand cells were seeded into 12 well plates and incubated for 24 h. Medium was replaced with fresh culture medium containing 3 μg/ml of avelumab-IR700 or diluted IR700 dye and incubated for 6 h at 37°C. After washing with PBS, PBS was added, and cells were irradiated with a red light-emitting diode (LED), which emits light at 670-710 nm wavelength (L690-66-60; Marubeni America Co., Santa Clara, CA, USA) at a power density of 50 mW/cm^2^ as measured with an optical power meter (PM 100, Thorlabs, Newton, NJ, USA). Cells were scratched 1 h after treatment. PI was then added in the cell suspension (final 2 μg/ml) and incubated at room temperature for 30 min, followed by flow cytometry. Each value represents mean ± standard error of the mean (s.e.m.) of five experiments.

### Animal and tumor models

All *in vivo* procedures were conducted in compliance with the Guide for the Care and Use of Laboratory Animal Resources (1996), US National Research Council, and approved by the local Animal Care and Use Committee. Six to eight week old female homozygote athymic nude mice were purchased from Charles River (NCI-Frederick, Frederick, MD). During the procedure, mice were anesthetized with isoflurane. In order to determine tumor volume, the greatest longitudinal diameter (length) and the greatest transverse diameter (width) were measured with an external caliper. Tumor volumes were based on caliper measurements and were calculated using the following formula; tumor volume = length × width^2^ × 0.5. Body weight was also measured. Mice were monitored daily for their general health. The presence of skin necrosis or toxicity attributable to the APC was evaluated with the observation of skin color and general health, including weight loss and appetite loss. Tumor volumes were measured three times a week until the tumor volume reached 2000 mm^3^, whereupon the mice were euthanized with inhalation of carbon dioxide gas.

### *In vivo* fluorescence imaging studies

H441 cells (2 × 10^6^) were injected subcutaneously in the right dorsum of the mice. Tumors were studied after they reached volumes of approximately 50 mm^3^. Serial ventral and dorsal fluorescence images of IR700 were obtained with a Pearl Imager using a 700 nm fluorescence channel before and 0, ½, 1, 2, 3, 4, 5, 6, 9, 12, 24, 48, 72, 96, 120, 144, and 168 hours after i.v. injection of 100 μg of avelumab-IR700 via the tail vein. Pearl Cam Software (LICOR Biosciences, Lincoln, NE) was used for analyzing fluorescence intensities. Region of interests (ROIs) were placed on the tumor and liver. ROIs were also placed in the adjacent non-tumor region as background (left dorsum and lower abdomen). Average fluorescence intensity of each ROI was calculated. TBRs (fluorescence intensities of target/fluorescence intensities of background) were also calculated (n = 10).

### *In vivo* NIR-PIT

H441 cells (2 × 10^6^) were injected subcutaneously in the right dorsum of the mice. Tumors were studied after they reached volumes of approximately 50 mm^3^. To examine the therapeutic effect of *in vivo* NIR-PIT on H441 cells, tumor-bearing mice were randomized into 4 groups of at least 10 animals per group for the following treatments: (1) no treatment (control); (2) 100 μg of avelumab-IR700 i.v., no NIR light exposure (APC i.v. only); (3) NIR light exposure only, NIR light was administered at 50 J/cm^2^ on day 1 and 100 J/cm^2^ on day 2 (NIR light only); (4) 100 μg of avelumab-IR700 i.v., NIR light was administered at 50 J/cm^2^ on day 1 after injection and 100 J/cm^2^ on day 2 after injection (NIR-PIT). Serial fluorescence images, as well as white light images, were obtained before and after each NIR light exposure (day 1 and day 2) using a Pearl Imager with a 700 nm fluorescence channel.

### Histological analysis

To detect the antigen-specific micro-distribution in the tumor, fluorescence microscopy was performed. Tumor xenografts were excised from mice without treatment, 24 h after injection of avelumab-IR700 (APC i.v. only) and 24 h after NIR-PIT. Extracted tumors were frozen with optimal cutting temperature (OCT) compound (SAKURA Finetek Japan Co., Tokyo, Japan) and frozen sections (10 μm thick) were prepared. Fluorescence microscopy was performed using the BX61 microscope with the following filters: excitation wavelength 590 to 650 nm, emission wavelength 665 to 740 nm long pass for IR700 fluorescence. DIC images were also acquired. To evaluate histological changes, light microscopy study was also performed using Olympus BX61. Extracted tumors were also placed in 10% formalin and serial 10 μm slice sections were fixed on glass slide with H&E staining.

### Statistical analysis

Data are expressed as means ± s.e.m. from a minimum of five experiments, unless otherwise indicated. Statistical analyses were carried out using GraphPad Prism (GraphPad Software, La Jolla, CA, USA). For multiple comparisons, a one-way analysis of variance (ANOVA) followed by the Bonferroni's correction for multiple comparisons was used. The cumulative probability of survival based on volume (2000 mm^3^) was estimated in each group with a Kaplan-Meier survival curve analysis, and the results were compared with use of the log-rank test. Student's t test was used to compare the treatment effects with that of control. *P*-value of < 0.05 was considered statistically significant.

## SUPPLEMENTARY VIDEO





## Abbreviations

ADCC: antibody dependent cell-mediated cytotoxicity
ANOVA: one-way analysis of variance
APC: antibody-photo-absorber conjugate
DIC: differential interference contrast
EGFR: epidermal growth factor receptor
FDA: Food and Drug Administration
H&E: hematoxylin and eosin
IR700: IRDye700DX
LED: light-emitting diode
mAb: monoclonal antibodies
NIR: near-infrared
OCT: optimal cutting temperature
PBS: phosphate buffered saline
PI: propidium iodide
PIT: photoimmunotherapy
PD-L1: Programmed cell death protein-1 ligand
PDT: photodynamic therapy
ROI: regions of interest
SDS-PAGE: sodium dodecyl sulfate-polyacrylamide gel electrophoresis
SUPR: super enhanced permeability and retention
TBR: target-to-background ratio
UVC: ultraviolet C
