# Supplementary material for: Near infrared photoimmunotherapy with avelumab, an anti-programmed death-ligand 1 (PD-L1) antibody
Source: Oncotarget. 2016 Oct 3;8(5):8807–17. doi: 10.18632/oncotarget.12410 (PMC5341755; doi:10.18632/oncotarget.12410)
Supplement: Supplementary file 1 [file oncotarget-08-8807-s001.pdf]

Near infrared photoimmunotherapy with avelumab, an anti-programmed death-ligand 1 (PD-L1) antibody

SUPPLEMENTARY VIDEO

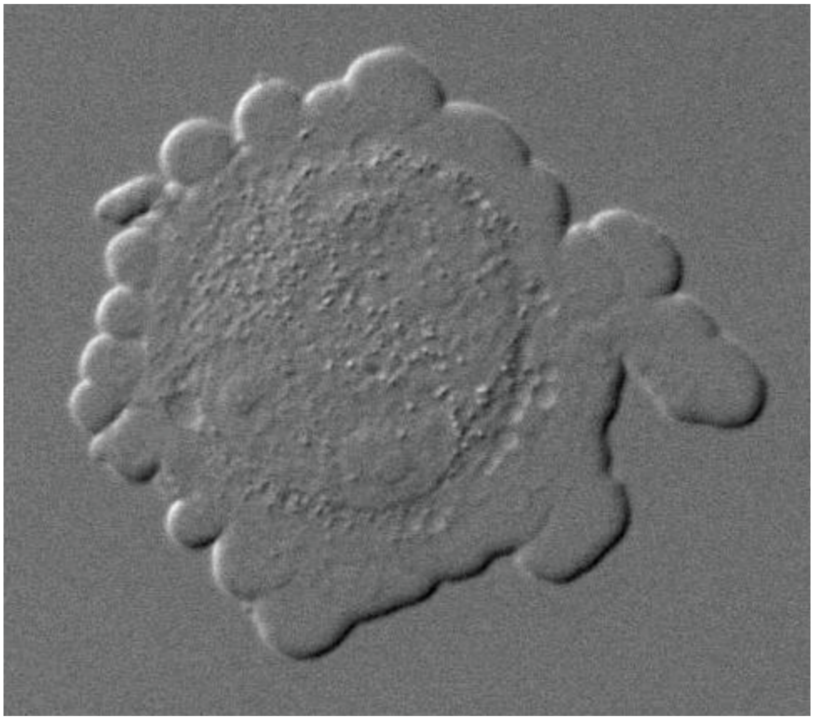

Supplementary Video S1: NIR-PIT effect for H441 cells. Immediately after exposure to excitation light cellular swelling, bleb formation, and rupture of vesicles representing necrotic cell death were observed.
